# Modeling Kelvin‐Helmholtz Instability at the High‐Latitude Boundary Layer in a Global Magnetosphere Simulation

**DOI:** 10.1029/2021GL094002

**Published:** 2021-10-07

**Authors:** A. T. Michael, K. A. Sorathia, V. G. Merkin, K. Nykyri, B. Burkholder, X. Ma, A. Y. Ukhorskiy, J. Garretson

**Affiliations:** ^1^ The Johns Hopkins University Applied Physics Laboratory Laurel MD USA; ^2^ Department of Physical Sciences and Center for Space and Atmospheric Research (CSAR) Embry‐Riddle Aeronautical University Daytona Beach FL USA

**Keywords:** Kelvin‐Helmholtz instability, geospace modeling, high‐latitude boundary layer

## Abstract

The Kelvin‐Helmholtz instability at the magnetospheric boundary plays a crucial role in solar wind‐magnetosphere‐ionosphere coupling, particle entry, and energization. The full extent of its impact has remained an open question due, in part, to global models without sufficient resolution to capture waves at higher latitudes. Using global magnetohydrodynamic simulations, we investigate an event when the Magnetospheric Multiscale (MMS) mission observed periodic low‐frequency waves at the dawn‐flank, high‐latitude boundary layer. We show the layer to be unstable, even though the slow solar wind with the draped interplanetary magnetic field is seemingly unfavorable for wave generation. The simulated velocity shear at the boundary is thin ($∼0.65\;R_{E}$) and requires commensurately high spatial resolution. These results, together with MMS observations, confirm for the first time in fully three‐dimensional global geometry that KH waves can grow in this region and thus can be an important process for energetic particle acceleration, dynamics, and transport.

## Key Points

- Global magnetohydrodynamic simulation of the magnetosphere captures unstable Kelvin‐Helmholtz waves at the high‐latitude boundary layer for the first time
- The growth of the surface waves occurs despite the stabilizing slow solar wind and draped magnetic field near the high‐latitude cusp
- The fastest growing wave mode resolved by the simulation is consistent with Magnetospheric Multiscale mission observations of the same event

## 1 Introduction

The Kelvin‐Helmholtz instability (KHI) is one of the important mechanisms controlling mass and energy transport between the solar wind and the magnetosphere. KHI generates boundary waves that propagate tailward along the magnetopause velocity shear layer (Farrugia et al., 1998). The vortices produced by the nonlinear instability facilitate plasma mixing, particle entry (Wing et al., 2014, and references therein), and loss (Sorathia et al., 2017).

The impact of KHI on the magnetosphere is particularly prominent at the low‐latitude boundary layer (LLBL), where the magnetic field is perpendicular to the plasma flow for northward or southward interplanetary magnetic field (IMF) orientations. This limits the stabilizing effect from the magnetic tension (Chandrasekhar, 1961; Miura & Pritchett, 1982). Analysis of the stability criteria for the KHI (Chandrasekhar, 1961; Miura & Pritchett, 1982) shows that wave growth can occur at the boundary layer during periods of both northward and southward IMF. Observations have confirmed this, however, Kelvin‐Helmholtz (KH) waves during southward IMF are intermittent (Hwang et al., 2011) and are more easily identified during northward IMF (Fairfield et al., 2000; Hasegawa et al., 2004) when reconnection is not the dominant process on the day‐side.

The IMF orientation, though, is rarely purely northward or southward. An oblique IMF alters the draped magnetic field at the magnetopause, potentially modifying the location that is most unstable to the KHI (Henry et al., 2017). Much less is known about the high‐latitude boundary layer due to the need for observations in a polar orbit and the quickness with which spacecraft pass through the region. The strong magnetic field in the lobes and the presence of a component of the magnetic field parallel to the flow are thought to make the high‐latitude boundary layer more stable to the growth of the KHI. Haaland et al. (2019) observed the equatorial dawn side magnetopause thickness to be ∼1400 km. While the high‐latitude boundary layer is most likely thicker (Panov et al., 2008), it is still difficult to resolve in global simulations. Observations of magnetic field fluctuations near the polar cusp, however, provide indications that KH waves could propagate at high‐latitudes (D'Angelo, 1973; D'Angelo et al., 1974; Potemra et al., 1978). Linear theory, presented by Miura and Pritchett (1982), suggests that if a strong enough velocity shear is present, the high‐latitude cusp boundary could be subject to the KHI even with a parallel component of the magnetic field. The polar orbit of Cluster has provided an opportunity to study the high‐latitude region. Recent observations have shown the growth of KH waves at high latitudes near the northern hemispheric cusp when the IMF has a strong dawnward component (Hwang et al., 2012; Ma et al., 2016). The analysis of Hwang et al. (2012) showed that while the velocity shear at the high‐latitude was reduced relative to the LLBL, the layer was still KH unstable at the location of minimum magnetic shear. Additionally, the propagation direction was perpendicular to both draped IMF and geomagnetic fields, minimizing the stabilizing effect of the magnetic field.

In conjunction with observations, numerical simulations can probe why KH can or cannot form at certain latitudes. Local 2D simulations allow in‐depth analysis of the nonlinear growth, capturing the formation of a thick LLBL, widened by turbulence and KH waves (Manuel & Samson, 1993; Matsumoto & Seki, 2010), where magnetic reconnection within the vortices can lead to mass transfer across the boundary (Ma et al., 2017; Nakamura et al., 2017; Nykyri & Otto, 2001). Recently, global 3D simulations have illuminated the properties and impact of surface waves on the global magnetosphere (Claudepierre et al., 2008; Guo et al., 2010; Li et al., 2013; Merkin et al., 2013). Claudepierre et al. (2008) used the Lyon‐Fedder‐Mobarry (LFM) model (Lyon et al., 2004) with detailed spectral analysis to reveal two bands of KH oscillations at the LLBL which can transport and energize radiation belt electrons in the dusk sector. Merkin et al. (2013) showed that a double vortex sheet can form at the LLBL and that the entire magnetopause was globally unstable, including at the high‐latitude boundary layer, as surface waves driven by the KHI after the terminator were coupled to global body modes. These works, performed primarily with northward or southward IMF conditions, indicate the 3D nature of the KHI at the magnetopause. No global simulation, however, has resolved KH waves at high latitudes and therefore has limited our ability to determine the latitudinal extent of KH waves.

In this study, we use a global 3D model of the magnetosphere to demonstrate that the high‐latitude boundary layer can indeed be unstable to KHI growth under dusk‐dawn IMF conditions. We focus on an event when MMS observed quasi‐periodic, low‐frequency waves interpreted as KHI by Nykyri et al. (2021). MMS was at the dayside, dawn sector, high‐latitude magnetopause, with GSM coordinates of $\left(x,\;y,\;z\right)\approx \left(1,-10,-6\right)R_{E}$. The IMF was highly inclined, with a strong dawnward component, similar to Hwang et al. (2012), however, the magnetosphere was driven by slow solar wind ($n_{sw}∼15{cm}^{-3}$, $V_{sw}∼300$km $s^{-1}$). This reduces the velocity shear and increases the compressibility at the boundary layer, which creates conditions that are not conducive to KHI growth, especially at high latitudes with lower velocity shear and stabilizing field along the layer (Miura & Pritchett, 1982; Pu & Kivelson, 1983; Walker, 1981). We demonstrate, however, that given sufficiently high resolution, our global MHD simulation of this event exhibited unambiguous KH fluctuations thus confirming the interpretation of the MMS observations by Nykyri et al. (2021).

## 2 Model Description

In this study, we use the newly developed Grid Agnostic MHD with Extended Research Applications (GAMERA) MHD model (Sorathia et al., 2020; Zhang et al., 2019). GAMERA preserves the core numerical philosophy of its predecessor LFM code (Lyon et al., 2004), including high‐order spatial reconstruction and geometric flexibility through the use of arbitrary non‐orthogonal grids, but also makes significant computational advances optimizing the code for modern supercomputers (Sorathia et al., 2020). GAMERA utilizes a warped spherical grid with higher resolution where necessary, in particular, around the dayside magnetopause. The results presented here are from a simulation that uses a grid with 192×192×256 cells in the radial, polar, and azimuthal directions (the spherical symmetry axis coincides with the Solar Magnetic, SM, *X*‐axis). This grid corresponds to the highest resolution LFM grid and has ∼ 1,000 km resolution across the high‐latitude boundary layer near the position of MMS. GAMERA utilizes a high‐order numerical scheme with a seventh‐order spatial reconstruction which has increased resolving power (Zhang et al., 2019). LFM at this resolution has been used extensively to study both KHI at the magnetopause (Merkin et al., 2013) and mesoscale plasmasheet convection (Wiltberger et al., 2015; Merkin et al., 2019) which are equally hard to resolve in a global magnetosphere simulation.

The storm time Sym−H index for the event was predominantly positive leading up to the event and was approximately 0 during the period of MMS observations of quasi‐periodic fluctuations at the boundary layer. Therefore, we assumed the ring current does not play an important role in this event, and GAMERA was not coupled to the Rice Convection Model (RCM) (Toffoletto et al., 2003).

GAMERA is used to model the magnetosphere from 25 $R_{E}$ at the subsolar point to 300 $R_{E}$ down the magnetotail. The model has a spherical inner boundary at 2 $R_{E}$. The MHD solution along the inner boundary is coupled to a two‐dimensional, integrated ionospheric model, the RE‐developed Magnetosphere‐Ionosphere Coupler/Solver (REMIX). REMIX is a rewrite of the MIX code (Merkin & Lyon, 2010) that solves the ionospheric Ohm's law given the MHD field‐aligned currents and a tensor of height‐integrated conductivities in the ionosphere, computed using a quasi‐empirical model including both solar irradiance and precipitation contributions (Fedder & Lyon, 1995; Zhang et al., 2015). This allows for magnetospheric current closure and informs the MHD simulation of the motion of magnetic field lines at the inner boundary. The solar F10.7‐cm flux index was set to 89.9, the daily flux density during the event taken from the OMNI data set. REMIX used a uniform grid with 0.5° resolution in both latitude and longitude. The low latitude boundary was set to 45° magnetic latitude and the coupling frequency between the two models was set to be every 5 s.

The GAMERA‐REMIX geospace model is solved in the SM coordinate frame and is driven by solar wind data taken from the OMNI database at 1‐min resolution. Data gaps are linearly interpolated to provide continuous boundary conditions for the simulation. In this study, the IMF is assumed to consist of planar fronts with the IMF $B_{X}$ component set to 0. The MHD simulation was started at 12 UT on February 25, 2016 and preconditioned with real solar wind for almost 9 h before the period of interest between 18:55 and 20:05 UT. The three‐dimensional electromagnetic fields and plasma solution generated by the simulation are saved at a cadence of 10 s. This results in a Nyquist frequency of 50 mHz which allows us to capture the evolution of the 7–10 mHz fluctuations observed by MMS.

## 3 Surface Wave Analysis

**Figure 1:**
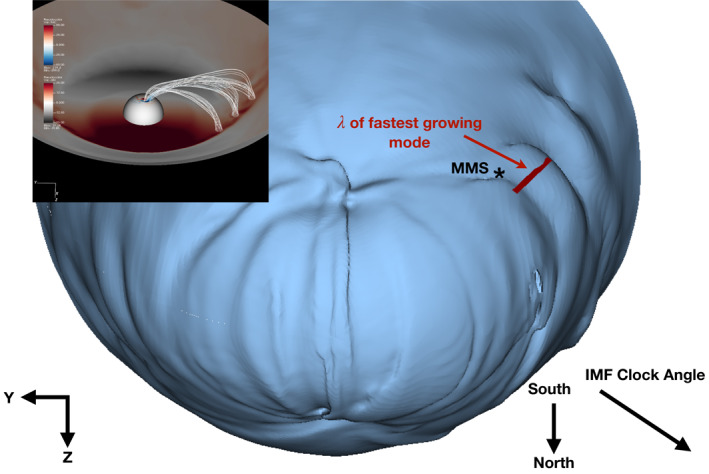
An overview of the simulated magnetosphere at 19:15 UT, February 25, 2016, where the *Z*‐axis is flipped. The iso‐surface is defined by a plasma density of *n* = 15 ${cm}^{-3}$. The location of MMS during the event is denoted by the asterisk. The red line is 4.88 $R_{E}$ long, for reference. The corresponding IMF clock angle is shown in the bottom right. The inset shows the residual magnetic field in the equatorial plane, with the dipole field removed. Regions where the field is compressed are in red. Magnetic field lines near the equatorial boundary layer are in white along with the field‐aligned currents at the spherical inner boundary surface.

An overview of the simulation is shown in Figure 1. The density iso‐surface was selected to best match the location where the magnetic field changes topology from closed, with both magnetic foot points seeded at the inner boundary, to open. Surface waves can clearly be seen in the vicinity of MMS. These waves extend over a wide range of latitudes and propagate tailward along the magnetopause. Within the Supporting Information S1, we provide a video detailing the dynamic behavior of the waves. The waves are intermittent with similar waves forming in the dayside, dusk sector of the northern hemisphere. This is consistent with the results of Hwang et al. (2012) who observed KH wave growth on the dusk side in the northern cusp during strong dawnward IMF conditions, similar to the ones here. The surface waves on the magnetopause form in the two quadrants opposite to the IMF clock angle where the draped IMF field does not have a component along the magnetopause providing a strong parallel magnetic field that stabilizes the boundary layer to KHI growth. This behavior is also seen in the supplemental video in the Supporting Information S1. The field lines, shown in the inset of Figure 1, map to the magnetospheric side of the LLBL and can be seen to be twisted within the surface waves, indicating an influence of the shear layer across the high‐latitude boundary layer. While it will not be discussed here, the twisted field lines are expected to carry field‐aligned currents and generate auroral structures associated with KH waves (Johnson et al., 2021).

**Figure 2:**
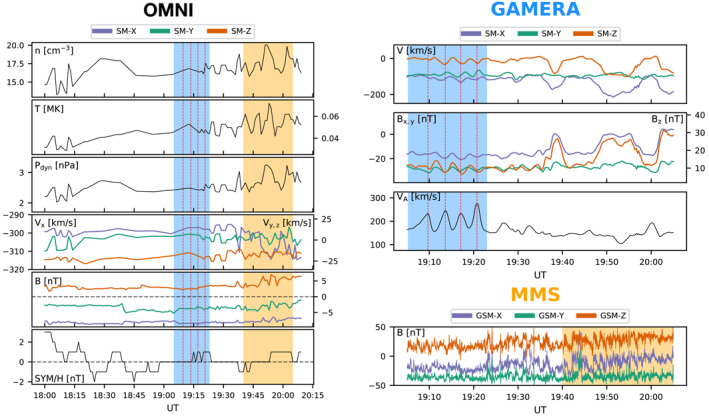
The left panel presents the solar wind conditions from the OMNI database during the event on February 25, 2016. The top right panel is a time series extracted from the simulation at the location on the magnetopause closest to the MMS position. The blue region highlights a period where surface waves were visible in the solution, the peaks occur at the red dashed lines. The bottom right panel presents magnetic field observations from MMS probe 3. Quasi‐periodic fluctuations were observed within the orange shaded region.

The solar wind conditions from the OMNI database, MMS observations, and simulation time series during the event are presented in Figure 2. The IMF upstream of the bow shock has a dominant radial, anti‐sunward, component with $B_{X}∼8$ nT. The cone angle of the IMF has been shown to affect the occurrence rate of KH waves at the LLBL, due to the presence of the collinear radial field and velocity shear for low cone angles (Kavosi & Raeder, 2015). Quasi‐periodic waves were observed in the z and x components of magnetic field from MMS probe 3 from 19:40 to 20:05 UT. The features occur nine times between 19:47 and 19:57, resulting in 10 mHz fluctuations in the maximum variance direction. During this period, Nykyri et al. (2021) also show lower density electrons in the MMS data exhibit the “faster‐than‐sheath” feature often interpreted as rolled‐up KH vortices (Hasegawa et al., 2006; Takagi et al., 2006).

The OMNI solar wind conditions were also used to drive the global MHD simulation. Large data gaps occurred between 18:15 and 19:20 UT, resulting in the simulation to be driven during this period mostly by smooth, interpolated, and predominantly steady solar wind conditions. The IMF in the simulation used in this study is planar, neglecting the large $B_{X}$. Since the $B_{Y}$ and $B_{Z}$ components are amplified across the bow shock by a factor of ∼4, the role of the strong $B_{X}$ impacting the magnetosphere is significantly diminished. Still, combined with the significant data gaps of solar wind data during the event, the conditions driving the magnetosphere in GAMERA are sufficiently different from the actual solar wind impinging MMS that the surface waves most likely will occur at different times and somewhat different locations than observed by MMS. We therefore do not provide a direct comparison to MMS and instead select a period where the surface waves are apparent in the simulation for a qualitative comparison of the stability of the boundary layer to KH growth.

Shown in the top right panel of Figure 2, the surface waves in the simulation are the most prominent between 19:05 and 19:22 UT as fluctuations can be seen in all plasma parameters. The waves occur at a frequency of ∼4.5 mHz. This is lower than the observed frequency by a factor of 2, which possibly suggests that the wavelength of the fastest growing mode, which scales with the shear layer thickness (Miura & Pritchett, 1982), is still under‐resolved in the simulation. Additionally, the waves are linear and do not have the vortex structure exhibited in the MMS data. The waves occur during a gap in the OMNI data, driven by relatively steady, interpolated solar wind. The waves are therefore not in response to rapid variations of the solar wind dynamic pressure. To determine whether the waves are driven by reconnection, we examined the magnetic field topology near the location of MMS to find the occurrence rate of flux transfer events (FTEs). From Movie S2, FTEs occur near the MMS location every ∼10 min or at a frequency of ∼1.6 mHz, which is too low to account for the 4.5 mHz waves seen on the magnetopause.

**Figure 3:**
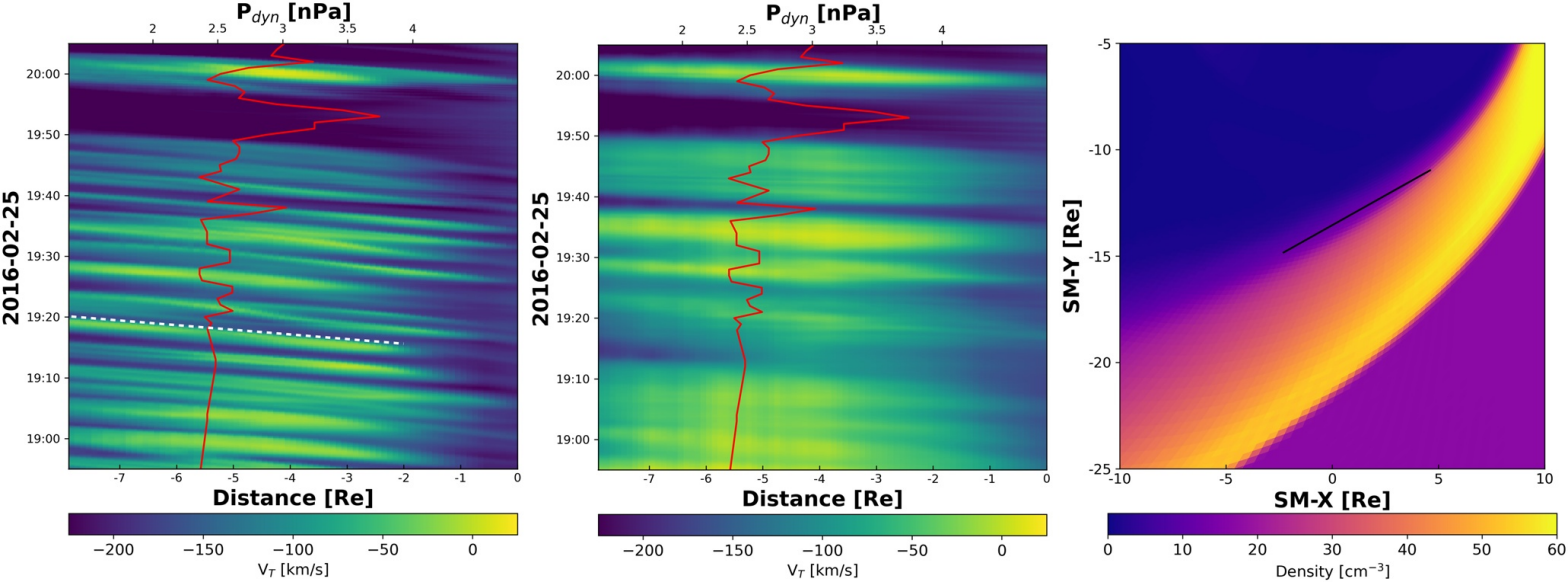
Spatiotemporal plots show contours of the tangential velocity, $V_{T}$, along the magnetopause, indicated by the black line in the right‐hand panel. The panel shows the plasma density averaged over the event in the plane through the peak of the surface waves in the southern hemisphere. The dip angle of the plane is 255°, measured clockwise from the +*Z* axis. The x‐axis denotes distance along the magnetopause, beginning at the location closest to the nose. The left‐hand panel shows the results from this study. The middle panel shows results from the same simulation run using a grid that is twice as coarse in each dimension. For reference, the red curve is the solar wind dynamic pressure as it passes Earth.

We estimate the phase speed of the waves using a similar technique employed by Claudepierre et al. (2008) and Merkin et al. (2013). The spatiotemporal evolution of the boundary layer is presented in Figure 3. Surface waves appear as sloped lines, forming pairs of yellow and blue stripes, as the crests and valleys of the wave propagate tailward along the boundary layer. In the left‐hand panel of Figure 3, surface waves can be seen throughout the event except during periods of strong dynamic pressure and inward movement of the magnetopause. To emphasize this point, the middle panel shows the results from the same simulation run with a grid that is coarser by a factor of 2 in each dimension, decreasing the cell resolution to 0.35 $R_{E}$ at the high‐latitude boundary layer. In this coarser simulation, the boundary layer is not resolved and no surface waves appear during the period from 18:50 and 19:20 UT. Additionally, the tangential velocity along the magnetopause is directly correlated to the motion of the magnetopause in response to the variation in the solar wind dynamic pressure. The phase speed of the surface waves is calculated by taking the slope of the structures in the figure. The phase speed estimated from the slope of the white dashed line in Figure 3 is $V_{ph}\approx 140$ km/s. This is comparable to the value obtained by Nykyri et al. (2021) from MMS data using plasma properties and magnetic field on either side of the boundary layer in the unperturbed magnetosheath and magnetosphere between 18:55 and 20:05 UT. The wave phase speed can vary as a function of time and distance along the boundary layer (Merkin et al., 2013), however, during the period of steady solar wind conditions within the simulation before 19:20 UT, the phase velocity appears to be uniform across the waves.

**Figure 4:**
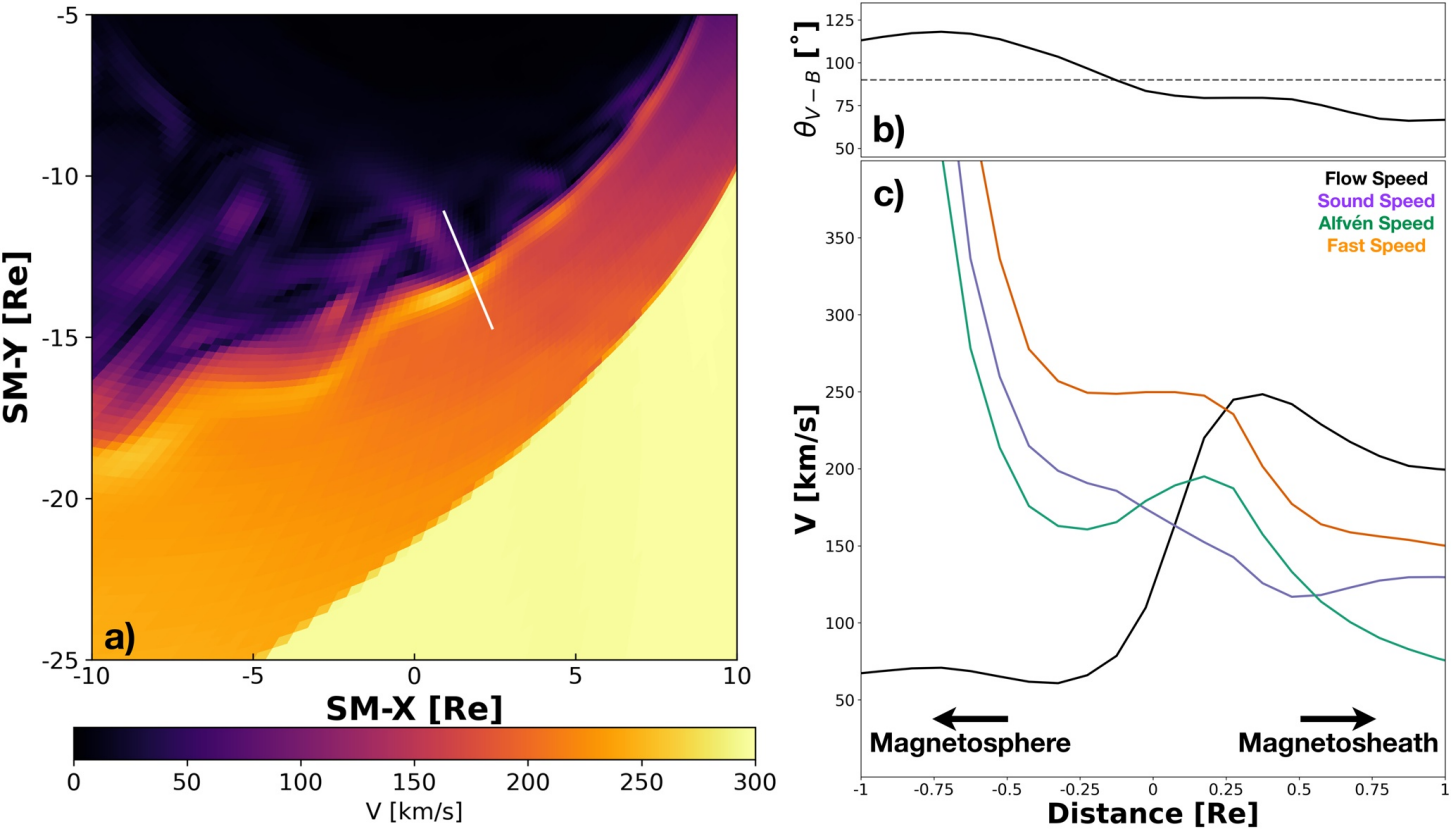
Contours of the plasma speed contained within the same plane as Figure 3 are shown in panel (a) taken at 19:15 UT in the simulation. Profiles of the angle between the velocity vector and magnetic field direction (b) as well as plasma flow speed and relevant MHD modes (c) across the high‐latitude boundary layer are shown. The white line in panel (a) denotes where the profiles were extracted.

Figure 4 presents the profile of the plasma flow parameters across the boundary layer at 19:15 UT. The shear layer is defined by the locations of minimum and maximum flow speed, resulting in a total velocity shear of $V_{0}\approx$184 km/s and a boundary layer thickness of $\Delta =0.65R_{E}$. With a cell resolution of $0.17R_{E}$ at the high‐latitude magnetopause, we have just under four cells across the boundary layer, possibly still under‐resolving the velocity shear. The magnetic field is almost completely perpendicular to the velocity shear, with an average angle of $\theta_{VB}\approx 87.5{}^{\circ}$ across the boundary layer. The parallel component that provides a stabilizing effect on the boundary layer to KHI growth is small, resulting in a nearly transverse case and a boundary that is more unstable to KH waves.

To determine whether the high‐latitude boundary layer is KH unstable, we calculate the normalized wave number for the fastest growing wave mode we can resolve and compare directly to the linear theory presented by Miura and Pritchett (1982). The wavelength of the waves can be computed from the phase velocity and wave frequency:

(1) $$\lambda =\frac{V_{ph}}{f}=\frac{140\;km/s}{4.5\;mHz}\approx 4.88R_{E}.$$

The normalized wave number can then be calculated as:

(2) $$k\Delta =\frac{2\pi \Delta }{\lambda }=2\pi \frac{0.65R_{E}}{4.88R_{E}}\approx 0.84.$$

For reference, the wavelength is compared to the peaks of the waves in Figure 1 and shows good agreement. This wavelength also agrees well with 3–5$R_{E}$ estimate from Nykyri et al. (2021) who utilized both MMS data and local 2D KHI simulations. The compressibility of the plasma also can provide a stabilizing effect on the KHI. Miura and Pritchett (1982) characterize this effect with the compressibility factor, or the inverse ratio of the shear velocity to the fast mode speed, $M_{f}^{-1}=V_{f}/V_{0}$. In the simulation, the compressibility factor across the boundary layer in Figure 4 is $M_{f}^{-1}\approx 1.33$, using the average fast mode speed across the shear layer. The linear dispersion relation for the transverse case is presented in Figure 3 of Miura and Pritchett (1982). There is no curve corresponding to compressibility factor of 1.33, however, we can bound the normalized wave number from linear theory using the curves $M_{f}^{-1}=1.2$ and $M_{f}^{-1}=1.5$. Taking the location of the fastest growing mode on each curve, the normalized wave number from linear theory should be between kΔ∼0.84−0.88. This is in good agreement with the normalized wave number from the simulation and confirms that the boundary layer is unstable to KHI growth. Comparison of the boundary layer at higher and lower latitudes is presented in Figure S1. Due to the complex, 3D geometry at the high latitude extent of the waves, it is difficult to apply the analysis of Miura and Pritchett (1982) to confirm the local stability of the layer. The amplitudes of the waves, however, decrease at higher latitudes as the magnetic field component parallel to the flow shear increases. Reconnection can occur in weak KHI (Nakamura et al., 2006), therefore, despite the lower wave amplitude, is still important for plasma transport even at these latitudes.

## 4 Conclusions and Discussion

We have performed high‐resolution global simulations of the magnetosphere with realistic driving conditions during an event on February 25, 2016 that coincided with MMS observations of quasi‐periodic fluctuations in the dawnside southern hemispheric cusp. We have investigated the stability of the high‐latitude boundary layer to KH waves under these conditions, for the first time. In comparison to the linear theory of Miura and Pritchett (1982), we find that the high‐latitude boundary layer is unstable to the KHI and surface waves form on the magnetopause near the vicinity of MMS during a period when the magnetosphere was driven by steady solar wind conditions in the simulation. During this event, we find that the high‐latitude boundary layer has a width of $0.65R_{E}$ and a velocity shear of 184 km/s. Surface waves form on the magnetopause at the location where the velocity shear is nearly perpendicular to the magnetic field and there was no parallel component to stabilize the boundary. In the simulation, the waves occur with a frequency of 4.5 mHz and travel with phase velocities near 140 km/s. This results in a normalized wave number that is consistent with linear theory for compressible MHD KHI growth.

KHI growth occurred despite the magnetosphere being driven by slow wind. With solar wind velocities around ∼300 km/s, the velocity shear was almost 200 km/s. This is consistent with MMS observations during the event. Hwang et al. (2012) also reported a similar velocity shear when Cluster was in the northern high‐latitude cusp during a separate event where KH waves were observed also with frequencies between 1.1 and 7.5 mHz. Polar cusp turbulence would increase the boundary layer thickness (Sandahl, 2002; Savin et al., 2002, 2005), potentially impacting the high‐latitude extent over which KH waves can form in the simulation. Even with the absence of the turbulent boundary layer, the simulation provides good agreement with the data.

To our knowledge, this study presents the first global magnetosphere simulation of high‐latitude KHI. This is achieved due to the low numerical dissipation in our model needed to resolve the boundary layer. As seen in Figure 3, if the boundary layer is not resolved, the increased width will suppress wave growth stabilizing the boundary to the KHI. Even with 1,000 km resolution, the high‐latitude boundary layer is still probably under‐resolved resulting in the waves to remain linear without exhibiting the nonlinear structure observed by MMS. An even higher resolution would decrease the width of the boundary layer, allowing for higher frequency waves to grow that would provide a closer agreement to the ≈10 mHz waves in the MMS data. This marks the challenge of capturing high‐latitude KH waves in global simulations and the importance of high numerical accuracy in resolving the boundary layer to study mass and energy transfer between the solar wind and magnetosphere.

## Supporting information

Supporting Information S1

Movie S1

Movie S2

## Data Availability

The level 2 flux gate magnetometers (Russell et al., 2016) data from MMS can be obtained through the MMS Science Data Center at https://lasp.colorado.edu/mms/sdc/public/. The authors also acknowledge the use of the Space Physics Environment Data Analysis Software (SPEDAS) software used in the creation of the MMS figures in this study (Angelopoulos et al., 2019). The OMNI data set are available at https://cdaweb.gsfc.nasa.gov/index.html/ by selecting OMNI from the available spacecraft and then the 1 min resolution data. Simulation data used to create the figures are archived on Zenodo and available online via http://doi.org/10.5281/zenodo.5120556.
