# Supplementary material for: Modeling Kelvin‐Helmholtz Instability at the High‐Latitude Boundary Layer in a Global Magnetosphere Simulation
Source: Geophys Res Lett. 2021 Oct 7;48(19):e2021GL094002. doi: 10.1029/2021GL094002 (PMC9285077; doi:10.1029/2021GL094002)
Supplement: Supplementary file 1 — Supporting Information S1 [file GRL-48-0-s001.pdf]

Supporting Information for “Modeling Kelvin-Helmholtz Instability at the High-Latitude Boundary Layer in a Global Magnetosphere Simulation”

A. T. Michael[1], K. A. Sorathia[1], V.G. Merkin[1], K. Nykyri [2], B. Burkholder [2], X. Ma [2], A.Y. Ukhorskiy[1], J. Garretson[1]

[1]The Johns Hopkins University Applied Physics Laboratory, Laurel, MD, USA

[2]Department of Physical Sciences and Center for Space and Atmospheric Research (CSAR), Embry-Riddle Aeronautical University, Daytona Beach, FL, USA

Contents of this file

1. Figure S1

Additional Supporting Information (Files uploaded separately)

1. Captions for Movies S1 to S2

Movie S1. Animation of the nose view of the magnetopause, defined here by an isosurface where $n_p = 15$ cm^{-3}, as in Figure 1. The animation occurs between 19:05 - 19:25 UT within the simulation with the approximate location of the MMS spacecraft during that period denoted by an asterisks.

Movie S2. Animation of the V_Y velocity component of the velocity vector, in SM coordinates, in the Y= - 9.8 R_E plane. The location where MMS is located is denoted by an asterisks. The dynamic locations where the magnetic field changes topology from magnetospheric field lines to open field lines, and finally from open to the IMF in the magnetosheath are marked by the green

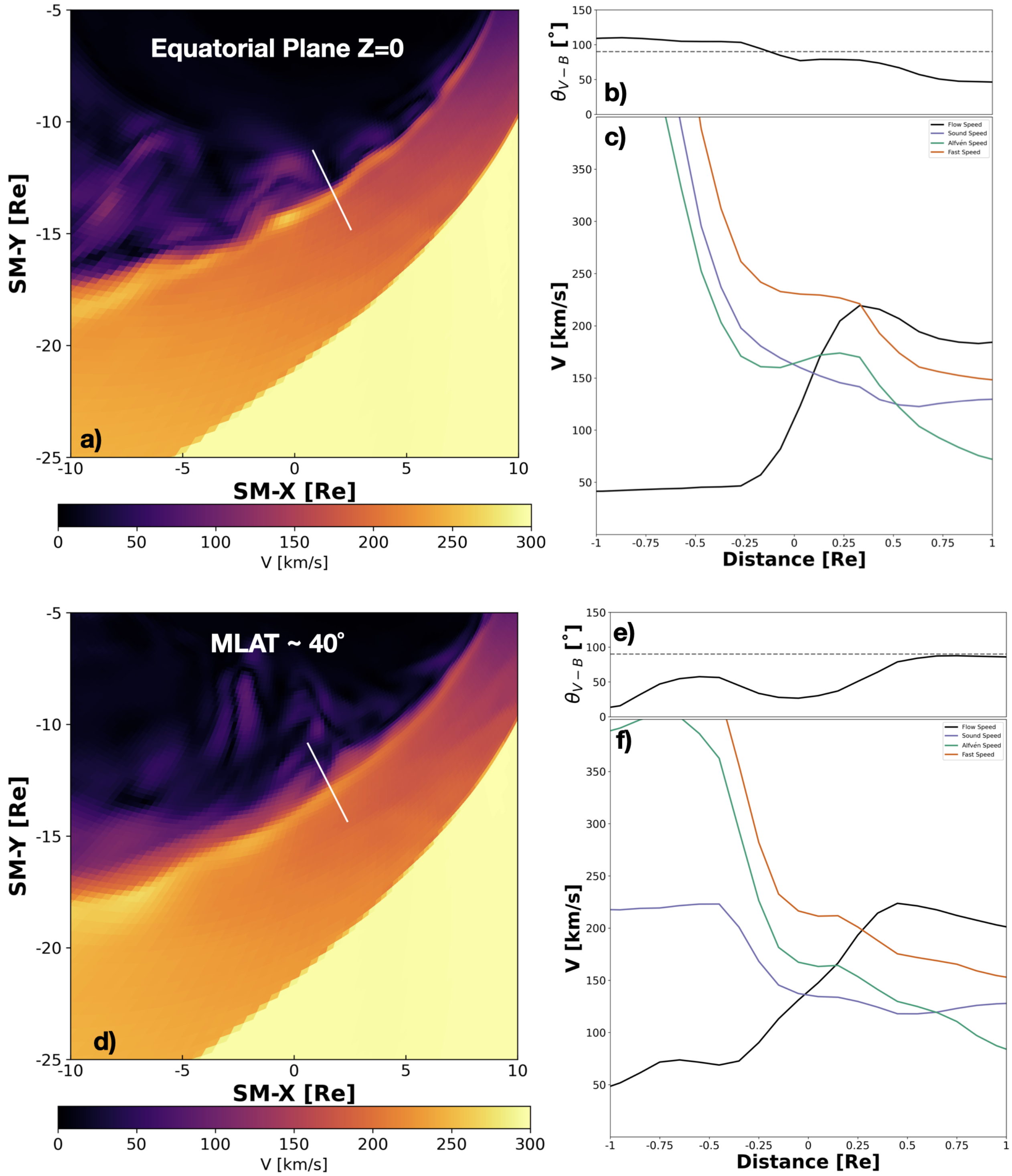

Figure S1. Similar to figure 4 detailing the boundary layer characteristics in the equatorial plane (Z=0RR_E) in panels a-c and for the plane at the high-latitude extent of the KH waves (magnetic latitude of $-40°$) above which KH waves are no longer form, panels d-f.

and yellow curves, respectively. Quantities are shown over the course of the event from 18:30 UT to 20:30 UT, 25 February 2016.
